# Longevity Aesthetics: A Conceptual Framework for Integrating Pro-Aging Medicine Into Surgical and Nonsurgical Practice

**DOI:** 10.1093/asjof/ojag088

**Published:** 2026-05-14

**Authors:** Diala Haykal, Foad Nahai

## Abstract

The prevailing paradigm in aesthetic medicine is the “antiaging” model, which focuses on correcting and/or reversing visible signs of aging. However, contemporary geroscience reconceptualizes aging as a malleable biological process driven by defined hallmarks, offering an opportunity to align aesthetic practice with the fundamental aging biology. This article explores the concept of “pro-aging” or “longevity aesthetics,” a framework that prioritizes tissue function, resilience, and biological integrity alongside phenotypic improvement. The skin is presented as an accessible model organ that reflects key hallmarks of aging and their clinical manifestations. Although conventional aesthetic interventions effectively address structural changes, they often do not target upstream biological drivers of tissue aging. Within this framework, certain surgical and nonsurgical modalities may theoretically support tissue resilience through modulation of inflammation, collagen remodeling, and microvascular health. Importantly, some of these strategies, such as biostimulatory injectables including calcium hydroxylapatite and poly-L-lactic acid, as well as combination treatment approaches, are already integrated into clinical practice, whereas others remain in the translational or research domain. Emerging concepts such as multi-omics, defined as the integration of genomics, transcriptomics, proteomics, metabolomics, and epigenomics, may further enable personalized and predictive approaches in the future. Longevity aesthetics, therefore, represents a conceptual evolution from correction to functional preservation. However, direct clinical evidence supporting its biological impact remains limited, and these concepts should currently be interpreted as hypothesis driven. Advancing this framework will require prospective studies integrating biomarkers with long-term clinical outcomes. Ultimately, this approach positions aesthetic medicine to contribute to translational geroscience, provided innovation is guided by methodological rigor and ethical transparency.

Level of Evidence: 5 (Therapeutic)

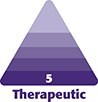

Aesthetic medicine and surgery have experienced exponential growth, driven by technological innovation and evolving societal demographics.^1^ Historically, this field has operated almost exclusively within an “antiaging” paradigm, where aging is framed as a series of cosmetic deficits to be reversed or concealed. Although this model has yielded significant technical progress and patient satisfaction, it has developed in parallel rather than in dialogue with fundamental science of aging.^1^

The emergence of geroscience, an interdisciplinary field dedicated to understanding the molecular and cellular mechanisms that drive aging, challenges this passive view. Geroscience posits that aging is not merely time's passage but a primary risk factor for functional decline, governed by conserved biological hallmarks.^2^ This paradigm shift invites a critical re-examination of aesthetic practice: Can interventions be assessed not only by their visual impact but also by their interaction with the biology of aging tissues? The proposed concept of “pro-aging medicine” or “longevity aesthetics” seeks to provide a complementary framework. This piece refers specifically to aesthetic longevity, focusing on the preservation of tissue function, resilience, and aesthetic integrity over time, and does not imply systemic disease modification, health span extension, or lifespan prolongation. It suggests aesthetic strategies could be proactively designed to support tissue health, enhance resilience, and improve functional responses to procedural stress, moving beyond purely reactive correction.

This article is presented as a Special Topic, providing a conceptual and translational overview of emerging regenerative and longevity-based approaches in aesthetic dermatology and surgery. Rather than a formal systematic review, it integrates current evidence with expert perspective to explore evolving frameworks and future directions in the field (Figure 1).

**Figure 1.**
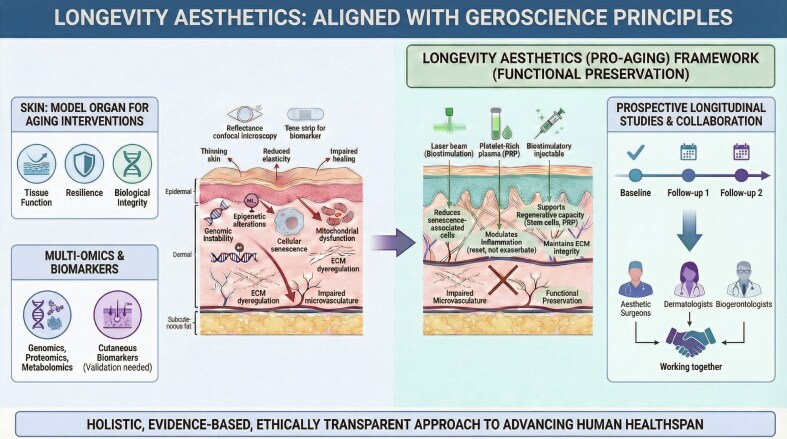
Longevity aesthetics: aligned with geroscience principles. This figure illustrates the shift from traditional antiaging approaches toward a regenerative, longevity-based framework in aesthetic medicine. It integrates biological hallmarks of aging with clinical interventions, including surgical procedures, biostimulatory injectables, and energy-based devices. It also highlights emerging tools such as artificial intelligence and multi-omics, defined as the integration of genomics, transcriptomics, proteomics, metabolomics, and epigenomics. Arrows represent interactions between biological processes and therapeutic strategies, emphasizing personalized and preventive care. During the preparation of this work, the authors used FigureLabs AI to create this figure. After using this tool, the authors reviewed and edited the content as needed and take full responsibility for the content of the figure.

## THE SKIN: AN ACCESSIBLE MODEL ORGAN FOR AGING AND INTERVENTION

The integumentary system provides a unique and practical interface for studying aging biology. The skin visibly and functionally manifests the classic hallmarks of aging, including genomic instability, epigenetic alterations, mitochondrial dysfunction, cellular senescence, and altered intercellular communication.^3,4^ Clinically, this translates to clinical signs well-known in aesthetic practice: dermal thinning, reduced elasticity, impaired barrier function, and delayed wound healing.^5^

Critically, these processes are not confined to the epidermis and dermis. The subcutaneous adipose layer, connective tissue, and microvascular networks, all critical to surgical and energy-based outcomes, undergo analogous age-related decline.^6^ For instance, adipose-derived stem cell exhaustion and senescent cell accumulation in subcutaneous tissue can impair volume retention and healing capacity.^7^ The skin's accessibility for noninvasive imaging (eg, reflectance confocal microscopy and optical coherence tomography) and repeated sampling (eg, transepidermal water loss, sebum secretion, and tape stripping for biomarkers) makes it an ideal organ for longitudinal study.^8^ This positions aesthetic practice, which routinely interfaces with this tissue, as a potential frontier for translational human aging research, provided claims are carefully validated.

## FROM ANTIAGING TO PRO-AGING: A PARADIGM SHIFT

The traditional antiaging model is inherently reactive and structural. Interventions, such as filler injection or facelift surgery, are employed to replace lost volume or resect loose skin, offering excellent correction but often after aging changes are established.^9^ This approach may not address the underlying biological “milieu” that led to the changes and could, in some cases of repetitive trauma or inflammation, inadvertently exacerbate tissue fragility. Table 1 provides a conceptual comparison of the 2 frameworks.

**Table 1.** Conceptual Comparison Between Antiaging and Pro-Aging (Longevity) Aesthetic Frameworks

Dimension	Traditional antiaging paradigm	Pro-aging/longevity aesthetics framework
Primary objective	Correction or concealment of visible age-related changes	Preservation of tissue function, resilience, and biological integrity
Concept of aging	Undesirable process to be reversed or minimized	Regulated biological processes that can be modulated
Timing of intervention	Reactive, initiated after phenotypic aging appears	Preventive, potentially earlier, guided by tissue condition and risk profiles
Primary outcome measures	Visual improvement, patient satisfaction, short-term safety	Functional tissue quality, durability of outcomes, biological markers (hypothesis driven)
Biological focus	Limited consideration of underlying aging mechanisms	Informed by hallmarks of aging (eg, inflammaging, senescence)
Role of procedures	Structural correction or surface modification	Biological modulation and tissue support (theoretical)
Evidence base	Established for aesthetic outcomes	Largely conceptual; requires longitudinal validation
Clinical limitations	May not address upstream aging drivers	Risk of overinterpretation without robust biomarkers
Ethical considerations	Focus on aesthetic benefit	Emphasis on evidence-based restraint and transparency

The pro-aging framework, informed by geroscience, proposes a complementary strategy. Its primary objective shifts toward preserving tissue function and resilience, conceptualizing aging as a modifiable process rather than an inevitable enemy.^10^ The timing of interventions would theoretically consider biomarkers of tissue health, potentially guiding earlier, preventative strategies.^11,12^ The role of procedures expands from structural correction to include biological modulation, such as laser therapy to reduce oxidative stress or platelet-rich plasma to support regenerative signaling.^13,14^

## PATHWAYS INTERSECTIONS: AESTHETIC MODALITIES AND AGING HALLMARKS

The scientific plausibility of longevity aesthetics rests on evidence that common aesthetic interventions interact with fundamental aging pathways.^3,15,16^

- Cellular senescence: Senescent cells accumulate in aged skin, secreting a pro-inflammatory, tissue-destructive senescence-associated secretory phenotype (SASP). Some energy-based devices (eg, specific laser wavelengths) and senolytic agents under investigation may selectively target these cells, potentially improving the tissue environment.^17-19^
- Inflammation: Chronic, low-grade inflammation is a key driver of aging. Many biostimulatory modalities (eg, microfocused ultrasound, radiofrequency, and certain injectables like calcium hydroxylapatite [CaHA] and poly-L-lactic acid [PLLA]) work by inducing a controlled, therapeutic wound-healing response. With the correct dose, this may reset local inflammatory tone rather than exacerbate it, promoting structured collagen remodeling rather than fibrosis.^20^
- Stem-cell exhaustion and regenerative capacity: Platelet-rich plasma and stromal vascular fraction delivery act to supply trophic factors and progenitor cells that support resident stem cells, potentially enhancing tissue repair and maintenance.^21^
- Extracellular matrix (ECM) dysregulation: Nearly all aesthetic treatments aim to boost collagen, elastin, and hyaluronic acid production. A pro-aging perspective views this not just as volume restoration but as active maintenance of ECM integrity, which influences mechanotransduction, cell signaling, and overall tissue resilience.^22^

## CLINICAL TRANSLATION: IMPLICATIONS FOR PRACTICE

### Surgical Practice

A longevity lens would redefine and optimize preoperative planning. Instead of viewing patient factors like poor skin quality or microvascular health as static risks, they become modifiable targets. Prehabilitation strategies could include growth factor–based topicals or nonablative lasers to improve epidermal barrier function and dermal collagen before surgery, potentially leading to improved outcomes, faster healing, and better scars. Postoperative, interventions could be tailored to support resolution of inflammation and promote optimal scar maturation.^23^

### Nonsurgical Practice as Functional Preparation

Energy-based and injectable treatments could be sequenced not just for immediate cosmetic benefit but to “condition” tissue for future procedures or to maintain functional capacity. For example, regular biostimulatory injectable treatments, such as hyperdilute CaHA or PLLA, may maintain dermal support and tissue quality; according to perioperative expert consensus, patients treated with these agents generally remain suitable surgical candidates when appropriate timing and anatomical planes are respected, potentially deferring or optimizing the need for more invasive interventions.^24-26^

Retrospective clinical reports and randomized trials in scar management indicate that energy-based devices, including low-level lasers, pulsed dye lasers, and fractional modalities, improve scar outcomes by modulating collagen remodeling and enhancing tissue pliability and vascularity. These qualitative improvements in wound structure and healing are thought to correlate with more favorable surgical tissue handling and potentially reduced operative challenges compared with untreated fibrotic tissue.^27-30^ Retrospective observations suggest tissues treated with certain biostimulatory agents may handle surgery better but prospective trials are needed.

### The Integrated Treatment Continuum

The most significant implication is the move toward a unified, lifelong aesthetic health plan. In this model, nonsurgical modalities maintain tissue biology and resilience during early aging, whereas surgical interventions are timed strategically based on biological need rather than chronological age alone. Postsurgical maintenance would then focus on preserving the result by supporting the biological health of the newly repositioned tissues.

Although several elements of this framework are already applicable in current clinical practice, particularly biostimulatory injectables, combination therapies, and strategies aimed at improving skin quality, this model primarily serves as a conceptual structure to guide a shift toward prevention, tissue quality, and long-term functional outcomes.

## TRANSLATIONAL GAPS AND ETHICAL IMPERATIVES

Despite its promise, longevity aesthetics remains a largely conceptual and hypothesis-driven framework, and critical translational gaps must be addressed before widespread clinical implementation. First, there is no consensus on which cutaneous biomarkers, such as epigenetic clocks, SASP factors, or advanced glycation end-products, reliably reflect local vs systemic biological age or respond to aesthetic interventions. Second, aesthetic research remains dominated by short-term studies assessing outcomes over 6 to 12 months, resulting in a lack of longitudinal data on how repeated procedures influence tissue biology over years. Finally, the field lacks standardized, clinically feasible tools to objectively measure endpoints, such as “tissue resilience” or “regenerative capacity” in clinical practice.

Clinicians therefore have an obligation to clearly distinguish between established cosmetic outcomes and theoretical biological benefits, ensuring patient autonomy through transparent communication regarding the speculative nature of many pro-aging claims.

Importantly, this framework should be interpreted along a continuum between current practice and future innovation. Several elements are already actionable today, particularly the use of biostimulatory injectables, combination therapies, and strategies aimed at improving tissue quality and resilience rather than solely correcting volume loss. These approaches are increasingly integrated into routine clinical practice and align with the principles outlined in this model. In contrast, other components, such as validated cutaneous biomarkers, multi-omics-guided personalization (referring to the integrated analysis of genomics, transcriptomics, proteomics, metabolomics, and epigenomics), and predictive modeling through artificial intelligence, remain largely within the translational research domain.

At present, clinicians are not expected to directly implement these advanced tools or engage operationally with specialized fields such as biogerontology. Rather, the value of this framework lies in guiding a conceptual shift: encouraging clinicians to adopt a more preventive, tissue-quality-focused, and biologically informed approach while remaining strictly grounded in evidence-based practice. As research evolves, these emerging tools may progressively become accessible and clinically relevant, but their integration should follow robust validation and clear demonstration of clinical utility.

## FUTURE DIRECTIONS

Future efforts must prioritize the development and validation of practical, clinically relevant cutaneous biomarkers that reliably reflect biological aging processes. This requires initiating prospective, longitudinal cohort studies that integrate multi-omics profiling with standardized aesthetic interventions to establish causal links and define durability. Furthermore, the field must establish clinically meaningful endpoints for “tissue health” and “resilience” that extend beyond subjective scales.

Ultimately, realizing the potential of longevity aesthetics depends on forging sustained interdisciplinary collaborations between aesthetic surgeons, dermatologists, and biogerontologists. By embracing this integrative approach with methodological rigor and ethical transparency, the aesthetic field can responsibly advance its practice and contribute meaningfully to the broader science of human health span.

## CONCLUSIONS

Longevity aesthetics represents a conceptual evolution in aesthetic medicine, aligning clinical practice with the principles of geroscience by shifting the focus from the correction of visible aging to the preservation of tissue function and resilience. The skin provides a uniquely accessible model to explore whether aesthetic interventions can interact with biological aging processes. Although several elements of this framework are already reflected in current clinical practice, the overall model remains largely conceptual and hypothesis driven. Its future clinical relevance will depend on rigorous validation, long-term data, and continued integration of biological insights into evidence-based aesthetic care.
